# Simultaneous Determination of Night Effective Constituents and Correlation Analysis of Multiconstituents and Antiplatelet Aggregation Bioactivity *In Vitro* in Chuanxiong Rhizoma Subjected to Different Decoction Times

**DOI:** 10.1155/2019/8970624

**Published:** 2019-11-22

**Authors:** Peihua Zhang, Linming Chen, Xiaoxiao Wang, Jinpei Chen, Shungui Xu, Ling Ye, Yixin Yao

**Affiliations:** ^1^The Affiliated People's Hospital of Fujian University of Traditional Chinese Medicine, Fuzhou 350122, China; ^2^Kangmei Pharmaceutical Co., Ltd., Puning 515300, China; ^3^Deyang Food and Drug Safety Inspection and Testing Center, Deyang 61800, China

## Abstract

Several effective constituents, such as vanillin, ferulic acid, senkyunolide I, senkyunolide H, coniferyl ferulate, Z-ligustilide, butylphthalide, senkyunolide A, and levistilide A, are unstable and possess mutual transformation relationships in Chuanxiong Rhizoma (CR). Traditional Chinese medicine mainly involves decoction, and the content of effective constituents and antiplatelet aggregation bioactivity (AAB) in CR may vary with different decoction time (10 min, 20 min, 30 min, 40 min, 50 min, and 60 min). Here, we showed that coniferyl ferulate and levistilide A were detected in CR material, but not in the decoction. The effective components possessed transformation and degradation in CR decoction of different times. The effective components and the strength of AAB at 10 and 20 minutes were the strongest, followed by 30–50 minutes, and 60 minutes were the weakest by analysis of SIMCA-PLS in CR decoction of different times. In the Pearson correlation analysis, there were correlations (*P* < 0.05) between effective components, which were ferulic acid and senkyunolide I (coefficient was 0.976), ferulic acid and senkyunolide H (coefficient was 0.972), senkyunolide I and senkyunolide H (coefficient was 0.982), senkyunolide A and butylphthalide (coefficient was 0.974), senkyunolide A and Z-ligustilide (coefficient was 0.947), and butylphthalide and Z-ligustilide (coefficient was 0.993). Effective components (ferulic acid, senkyunolide I, and senkyunolide H) and AAB were correlated and the Pearson correlation coefficients were respectively 0.965, 0.973, and 0.999. In the stepwise regression analysis, senkyunolide H and senkyunolide I were correlated with AAB (*P* < 0.05). Senkyunolide H (H) was positively correlated with AAB, senkyunolide I (I) was negatively correlated with AAB, and its expression was AAB = 1.187 *∗* H − 0.199 *∗* I − 0.422. These findings indicate that there are some correlations between effective components and AAB in CR.

## 1. Introduction

**Figure 1:**
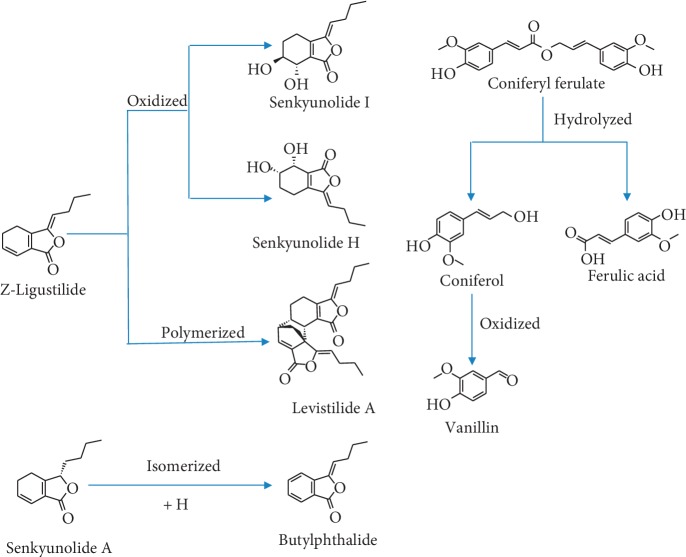
Conversion relation of nine effective constituents.

Chuanxiong Rhizoma (CR), the dried rhizome of *Ligusticum chuanxiong* Hort. (Umbelliferae), is one of the most commonly prescribed traditional Chinese medicines (TCM) for activating blood and removing stasis (effect of TCM) [1]. Recent studies have shown that CR and related Chinese patent drugs are prescribed for the treatment of cardiovascular and cephalagra diseases [2, 3]. The main constituents of CR include phthalides and phenolic acids. Phthalides include Z-ligustilide, senkyunolide A, senkyunolide I, senkyunolide H, and butylphthalide [4–6]. The effects of Z-ligustilide are vasodilatation and neuroprotective [7, 8]. Senkyunolide A, senkyunolide I, and senkyunolide H can inhibit the formation of thrombi, increase cerebral blood flow, and reduce cerebral vascular resistance [9–12]. Butylphthalide imparts neuroprotective effects [13–15]. Ferulic acid and coniferyl ferulate are the main constituents of phenolic acids [5, 6]. The effects of ferulic acid include antithrombosis and antiplatelet aggregation. Sodium ferulate is a common cardiovascular drug [16]. Coniferyl ferulate imparts antioxidant and vasodilatation effects [17]. Vanillin is the raw material or intermediate for many cardiovascular and cerebrovascular drugs (e.g., methyldopa) [18]. Traditional Chinese medicine mainly involves decoction. Most effective constituents of CR are unstable and easily decompose or transform. For example, Z-ligustilide readily oxidizes into senkyunolide I and senkyunolide H or polymerizes into levistilide A in air or with heating [19]. Coniferyl ferulate is easily hydrolyzed into ferulic acid and coniferol during extraction or when left to stand as a liquid [20]. Coniferol can be further oxidized to vanillin [21]. Senkyunolide A is unstable, and is easily oxidized and isomerized into butylphthalide [22] (Figure 1). Therefore, different decoction times could contribute to changes in the content of the active substances. The syndrome of blood stasis is strongly associated with thrombus-related diseases. Platelet aggregation is one of the direct causes of thrombosis. Antiplatelet aggregation bioactivity (AAB) is an index that reflects the dissipating blood stasis activity of CR [23]. Different decoction times could also contribute to changes in AAB of CR, resulting in differences in clinical efficacy.

To date, no study on the determination of multiconstituents and AAB in CR decoctions at different decoction times has been conducted. In this study, the ultraperformance liquid chromatography (UPLC) method was utilized to quantitatively determine the content of nine constituents (vanillin, ferulic acid, senkyunolide I, senkyunolide H, coniferyl ferulate, Z-ligustilide, butylphthalide, senkyunolide A, and levistilide A) in CR materials or decoctions at different decoction times, and the AAB was determined simultaneously. In different decoction time periods (10 min, 20 min, 30 min, 40 min, 50 min, and 60 min), the difference in effective constituents was analyzed by SIMCA-PLS and cluster analysis, and the difference of AAB was analyzed by Student–Newman–Keuls. The correlation between effective constituents and AAB was assessed using SIMCA-PLS, Pearson correlation analysis, and stepwise regression analysis. This study conducted a basic assessment of using CR in TCM.

## 2. Materials and Methods

### 2.1. Materials and Reagents

Acetonitrile (HPLC grade) was obtained from Fisher Corporation (Waltham, MA, USA). Adenosine-5-two sodium phosphate (C_10_H_13_N_5_Na_2_O_10_P_2_) was purchased from Sigma-Aldrich Co (St. Louis, MO, USA). Sodium ferulic acid was obtained from Shanghai Yuan Ye Biotechnology Co., Ltd. (Shanghai, China). Glacial acetic acid, dimethyl sulfoxide (DMSO), and three sodium citrate (two water) C_6_H_5_Na_3_O_7_·2(H_2_O) were of analytical grade and acquired from Guangzhou Chemical Reagent Factory (Guangzhou, China). Sodium chloride injection (0.9%, W/V) was purchased from Sichuan Cologne Pharmaceutical Co., Ltd. (Chengdu, China). CR decoction pieces (Product Batch No. 180802731) were manufactured by Kangmei Pharmaceutical Co., Ltd. (Guangdong, China) and deposited in the traditional Chinese Medicine Laboratory of Puning Production Base of Kangmei Pharmaceutical. Standards of vanillin, ferulic acid, senkyunolide I, senkyunolide H, coniferyl ferulate, Z-ligustilide, butylphthalide, senkyunolide A, and levistilide A (purities ≥ 98% by HPLC) were purchased from Chengdu Pufei De Biotech Co., Ltd. (Chengdu, China). Japanese big-eared white rabbits, male, weighing about 2.5 kg, were provided by Chengdu Dashuo Biotechnology Co., Ltd. (Chengdu, China).

### 2.2. Apparatus

BSA224S Precision electronic balance was purchased from Beijing Sartorius Scientific Instrument Co., Ltd. (Beijing, China). ZNHW-II Intelligent digital display electric hearting set was purchased from Gongyi Yuhua Instrument Co., Ltd. (Tianjin, China). Frontier™ 5000 Multi Pro multifunction centrifuge was purchased from OHAUS Company (Pine Brook, USA); RE-2000B rotatory evaporator was purchased from Shanghai Yarong Biochemical Instrument Factory (Shanghai, China). SC-2000 platelet aggregation instrument was purchased from Beijing Succeeder Technology Development Co., Ltd. (Beijing, China). KQ-500VDE double-frequency digital ultrasonic cleaning instrument was purchased from Kunshan Ultrasonic Instrument Co., Ltd. (Kunshan, China). UPLC was performed with an Agilent 1290 Infinity II system (Agilent, Palo Alto, CA, USA) equipped with binary pump solvent management system, online degasser, and autosampler.

### 2.3. Determination of Constituents by UPLC

#### 2.3.1. Condition of UPLC

The column was an Agilent Eclipse Plus C_18_ column (1.8 *μ*m, 50 mm × 2.1 mm, Agilent), and the column temperature was kept at 30°C. The flow rate was set at 0.3 mL·min^−1^. The injection volume was 2 *μ*L. The detection wavelength was set to 280 nm. 1% Acetonitrile was selected as mobile phase A, and glacial acetic acid (V/V) was selected as mobile phase B. The linear gradient elution of A was performed as follows: 18%–25% A at 0–4 min, 25%–46% A at 4–5 min, 46%–62% A at 5–9 min, 62%–72% A at 9–12 min, 72%–100% A at 12–15 min, and 100% A at 15–20 min.

#### 2.3.2. Preparation of CR Methanol Ultrasound Extraction

Each dried material was pulverized to 50 mesh. Approximately 0.5 g of pulverized powder was accurately weighed and then extracted with 50 mL methanol by ultrasound extraction (300 W of efficiency, 45 kHz of frequency) for 1 h, cooled to room temperature, and the final solution volume was adjusted to 50 mL with methanol.

#### 2.3.3. Preparation of CR Water Extraction

Each dried material was pulverized to 50 mesh. Approximately 0.5 g of pulverized powder was accurately weighed and then extracted with 50 mL pure water by decocting (100°C) for 10 min, 20 min, 30 min, 40 min, 50 min, and 60 min, cooling to room temperature, and the final solution volume was adjusted to 50 mL with pure water. The solution was centrifuged at 6,000 r/min for 20 min. The supernatant was collected and passed through a filter (0.22 *μ*m mesh size).

### 2.4. Determination of AAB

#### 2.4.1. Test Substance Solutions

The test substance solutions are citrate three sodium anticoagulant (3.2%, W/V), adenosine-5-two sodium phosphate solution (C_10_H_13_N_5_Na_2_O_10_P_2_; final concentration: 10 *μ*mol/L), and positive drug solution (sodium ferulate, final concentration: 2 mg/mL).

#### 2.4.2. Preparation Solution of CR Sample for AAB Analysis

Each dried material was pulverized to 50 mesh. Approximately 0.5 g of pulverized powder was placed into each of the 10 conical flasks, to which 50 mL of pure water was added. The solution was weighed and decocted for 10 min, 20 min, 30 min, 40 min, 50 min, and 60 min, to complement weightlessness. The extract was filtered with a gauze, centrifuged, and then subjected to vacuum filtration with a filter paper. The extract was concentrated to about 5 mL by decompressing (85°C, 15 min) and then transferred to 10 mL volumetric flasks. Pure water was added to volume and then shaken well. Approximately 1 mL of the extract was placed in a 10 mL volumetric flask, mixed with 1.5 mL of DMSO solution to dissolve, and then physiological saline was added to volume and shaken well. The solution was transferred to a centrifuge tube and centrifuged at 6,000 rpm for 20 min, and then the supernatant was collected.

#### 2.4.3. Preparation of Platelet-Rich Plasma (PRP) and Platelet-Poor Plasma (PPP)

Heart blood of the normal rabbits was collected and mixed with trisodium citrate solution as anticoagulant. The ratio of trisodium citrate solution and blood was 1 : 9. The anticoagulant and blood were mixed thoroughly by gently inverting the centrifuge tube.

The mixture was centrifuged at 800 rpm for 10 min twice. The upper plasma, which was the platelet-rich plasma (PRP), was collected. The lower layer of blood was collected from the first centrifugation and then centrifuged at 3,500 rpm for 10 min. The upper layer of the plasma was platelet-poor plasma (PPP).

#### 2.4.4. Determination of Platelet Maximum Aggregation Rate In Vitro and Calculation of Inhibition Rate

Approximately 280 *μ*L of PPP was added to three turbidimetric tubes, which each contained 10 *μ*L of the physiological saline, 10 *μ*L of the positive drug, and 10 *μ*L of the sample solution, as corresponding blank solutions. Approximately 280 *μ*L of PRP was placed into another three turbidimetric tubes, to which each was mixed with 10 *μ*L of physiological saline, 10 *μ*L of the positive drug, and 10 *μ*L of the sample solution, as corresponding sample solutions.

The platelet aggregation instrument was preheated to 37°C, and then 10 *μ*L of physiological saline was added to the blank solution and placed into the test hole to zero. The platelet aggregation rate was determined by preheating the platelet solution for 60 s and adding 10 *μ*L of adenosine-5-sodium diphosphate (C_10_H_13_N_5_Na_2_O_10_P_2_) solution. The inhibition rate of platelet aggregation of the samples or positive drugs was calculated using the following formula: platelet inhibition rate°=°(saline group maximum aggregation—sample or positive drug group maximum aggregation)/saline group maximum aggregation°×°100%. Parallel determination of each sample was performed 3 times [24].

### 2.5. Statistical Analysis

The results of effective constituents were analyzed by SIMCA-PLS and cluster analysis. The AAB results were expressed as the mean ± SD (standard deviation). The data were compared by one-way ANOVA followed by Student–Newman–Keuls with SPSS 19.0 software (Palo Alto, CA, USA). The differences were considered statistically significant when the different subsets in the subset of alpha = 0.05, conversely, the same subset was no significant difference. The correlation between constituents and AAB was analyzed by Pearson correlation analysis and stepwise regression analysis. In the Pearson correlation analysis, the differences were considered statistically significant when *P* < 0.05, and bivariate correlation coefficients were expressed. In stepwise regression analysis, when the value of *R*^2^ was larger, the model was more accurate. The differences of linear regression were considered statistically significant when *P* < 0.05. The relationship between independent variable and dependent variable could be expressed by expression.

## 3. Results and Discussion

### 3.1. Validation of Methodology

Development of the calibration curves: calibration curves were developed from the chromatographic peak area relative to the weights of vanillin, ferulic acid, senkyunolide I, senkyunolide H, coniferyl ferulate, Z-ligustilide, butylphthalide, senkyunolide A, and levistilide A. And limit of detection (LOD, S/N = 3) and limit of quantification (LOQ, S/N = 10) were calculated. The results are shown in Table 1.

The accuracy, repeatability, and stability (12 h) were evaluated by the peak areas of the nine constituents, with six samples in parallel, and they were expressed as RSD (%) within 5%. The result is shown in Table 2.

### 3.2. Analysis of Multiconstituents in CR Decoction at Different Decoction Times

**Figure 2:**
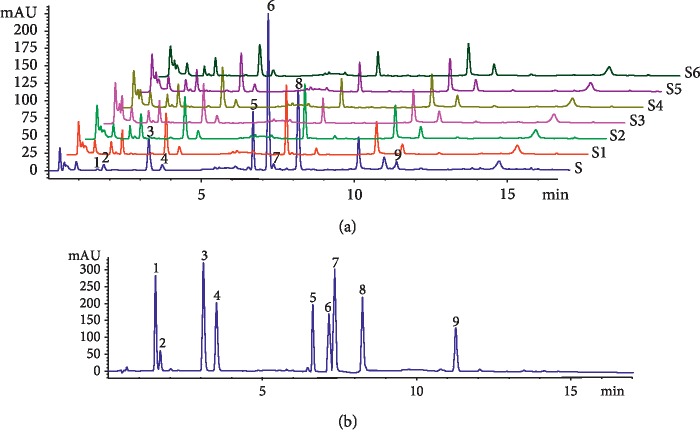
Chromatogram of samples and standard reference compounds of CR. (a) Chromatogram of samples in CR at various decoction times. S: methanol extraction; S1: 10 min; S2: 20 min; S3: 30 min; S4: 40 min; S5: 50 min; S6: 60 min. (b) Chromatogram of nine standard reference compounds. 1. Vanillin. 2. Ferulic acid. 3. Senkyunolide I. 4. Senkyunolide H. 5. Coniferyl ferulate. 6. Senkyunolide A. 7. Butylphthalide. 8. Z-Ligustilide. 9. Levistilide A.

**Figure 3:**
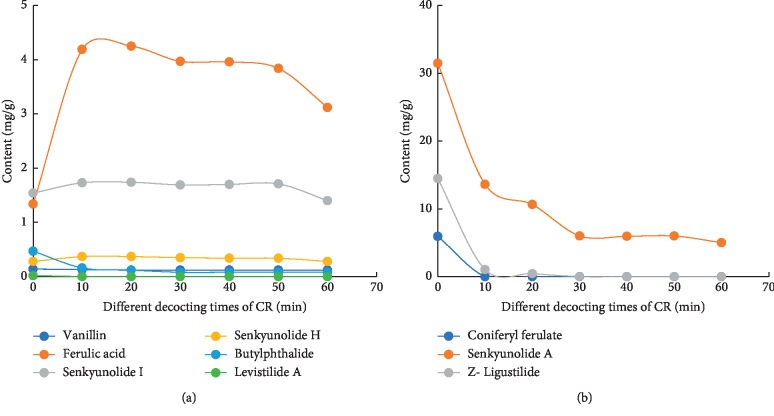
(a) Changes in vanillin, ferulic acid, senkyunolide I, senkyunolide H, and butylphthalide contents. (b) Changes in coniferyl ferulate, senkyunolide A, and Z-ligustilide contents.

When CR was extracted by the water in the extraction solvent, coniferyl ferulate can be hydrolyzed into ferulic acid and coniferol. At the same time, phthalides in CR are unstable and are easy to be degraded and transformed in the heating process. Therefore, in order to retain the nine active ingredients of CR to the greatest extent and which represent the ingredients contained of CR, 100% methanol was chosen as the extraction solvent, and ultrasonic extraction for 60 minutes was used to represent the nine active ingredients in CR [20, 21]. CR methanol extraction represented nine constituents of medicinal materials (0 min) (Figures 2 and 3 and Table 3). When the RSD value of the components was <5% using various decoction times, the contents did not change. The contents of vanillin decreased using 10–20 min decoction time but did not change with 20–60 min decoction time. The RSD value of content was 3.16% (*n* = 6) and was <5% using 10–60 min decoction time, and the content did not change using 10–60 min decoction time. Therefore, the content of vanillin did not further change using 10–60 min decoction time. The contents of ferulic acid increased with 10–20 min decoction time, peaking at 20 min, and then subsequently decreased with 20–60 min decoction time. The RSD value of content was 4.26% (*n* = 5) and was <5% with 10–50 min decoction time and the content did not change. Therefore, the content of ferulic acid initially stabilized, and then decreased with 0–60 min decoction time. This indicated that the increase in the content of ferulic acid stabilized with 10–50 min decoction time and was partially degraded within 50–60 min. The content of senkyunolide I increased with 10–20 min decoction time, and then peaked at 20 min. It then subsequently decreased within 20–30 min, then increased within 30–50 min, and finally decreased within 50–60 min. The RSD value of content was 1.11% (*n* = 5) and was <5% with 10–50 min of decoction, and then remained stable for 10–50 min. Therefore, the content of senkyunolide I initially stabilized for 10–50 min, and then decreased for 50–60 min. For senkyunolide H, the RSD value of content was 3.39% (*n* = 5) and was <5% within 10–50 min and did not change within 10–50 min. Therefore, the content of senkyunolide H initially stabilized, and then decreased from 10 to 60 min. Senkyunolide A decreased from 10 to 40 min, increased from 40 to 50 min, and then decreased from 50 to 60 min. The RSD value of content was 0.49% (*n* = 3) and was <5% from 30 to 50 min, and then remained unchanged from 30 to 50 min. Therefore, the contents of senkyunolide A initially decreased, then stabilized, and finally decreased from 10 to 60 min. The contents of butylphthalide decreased from 10 to 30 min, and then stabilized from 30 to 60 min. Therefore, butylphthalide content initially decreased and then remained unchanged within 10–60 min. The contents of Z-ligustilide within 10–30 min were 0 at 30 min. The contents of coniferyl ferulate and levistilide A were 0 at 10 min.

Trace amounts of coniferyl ferulate and levistilide A were undetectable at 10 min, indicating that these might have been degraded or not dissolved. Previous reports have shown that conifer ferulate readily hydrolyzes during decoction, and thus conifer ferulate degrades within 0–10 min. Levistilide A is a dimer of Z-ligustilide, and its chemical structure is more stable than Z-ligustilide, but Z-ligustilide content was higher at 0 min, and the degradation rate was higher within 0–10 minutes. Levistilide A was undetectable at 10 min, indicating that it might not have been dissolved. Based on the above results, the main constituents of CR were divided into two parts with different decoction times: the relative stable constituents and the unstable constituents. For the relative stable constituents, namely, ferulic acid, senkyunolide I, senkyunolide H, and vanillin, if we take the traditional decoction time 30 to 40 minutes as standard, then these four constituents were stable within 40 min during CR decoction and complied with the requirements of traditional decoction time. Ferulic acid, senkyunolide I, and senkyunolide H are active substances in CR, whereas vanillin requires further studies to confirm whether it is indeed an effective constituent. The unstable constituents included coniferyl ferulate, Z-ligustilide, senkyunolide A, butylphthalide, and levistilide A. The contents of these five constituents decreased or dissolved during decocting. The content of coniferyl ferulate was 0, but it could have undergone hydrolysis into ferulic acid during decoction, indicating that coniferyl ferulate is an indirect effective constituent. The dissolution rate of Z-ligustilide was low or easily degraded in water, but it can oxidize to senkyunolide I and senkyunolide H. However, as the oral bioavailability of Z-ligustilide was extremely low, the main metabolites *in vivo* included senkyunolide I, senkyunolide H, and *n*-butenyldenphthalide [24–26]. This indicated that Z-ligustilide is an indirect constituent of efficacy. Senkyunolide A also partially dissolved at 60 min and was easily converted to butylphthalide [21]; it could enter the bloodstream, indicating that senkyunolide A is a direct constituent. Butylphthalide showed similar bioactivity with CR efficacy and could enter the brain [13, 14]. It is a direct constituent during the decoction of CR. These findings indicated that the contents of constituents are different, and the effect on CR also differs with decoction time.

**Figure 4:**
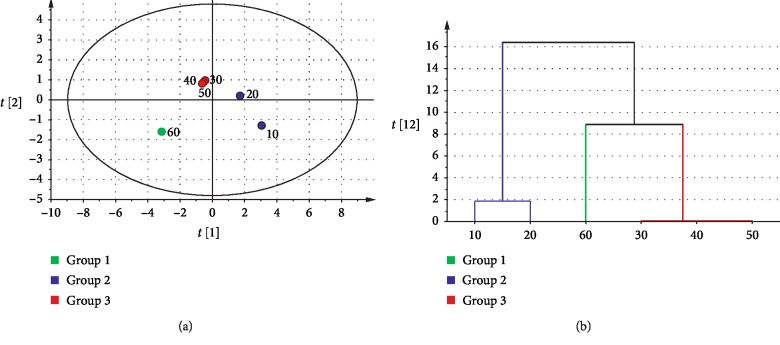
Results of statistical analysis by SIMCA-PCA and cluster analysis: (a) score scatter plot; (b) dendrogram.

The differences of effective components were comprehensively analyzed in 10–60 minutes of decocting time by SIMCA-PCA. Because the contents of coniferous ferulate and levistilide A were 0 and the difference was small with the contents of vanillin, the other effective components were carried out by SIMCA-PCA analysis and cluster analysis. The result of SIMCA-PCA indicated that the first two principal components were selected, *R*^2^*X* (cum) was 0.993 and *Q*^2^ (cum) was 0.959. Further combination of cluster analysis, 10 min and 20 min were one class; 30 min, 40 min, and 50 min were one class; and 60 min was one class (Figures 4(a) and 4(b)). The results showed that 20 min and 50 min were two demarcation points in decocting time of CR for 10–60 minutes. The richness of effective components can be indicated as 10 min, 20 min > 30 min, 40 min, 50 min > 60 min.

### 3.3. Analysis of AAB in CR Decoction Using Different Decoction Times In Vitro

**Figure 5:**
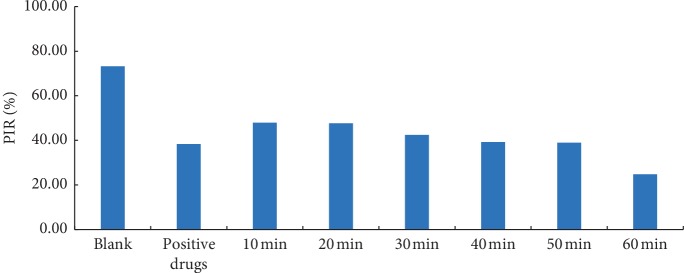
Platelet inhibition ratio of CR using different decoction times (*n* = 3).

Based on the results of antiplatelet aggregation, the calculated platelet inhibitory rate (PIR) was 10 min (47.94 ± 1.90%) > 20 min (47.63 ± 0.68%) > 30 min (42.38 ± 1.99%) > 40 min (39.20 ± 1.93%) > 50 min (38.93 ± 2.60%) > 60 min (24.76 ± 3.56%) (Figure 5). AAB is expressed in the platelet inhibitory rate, indicating that AAB of CR decoction was decreasing within 10–60 min.

One-way analysis of variance (ANOVA) was performed, and Student–Newman–Keuls (SNK) was selected. In the statistical analysis of SNK, there was no significant difference between the same subset and there was significant difference between the different subsets in the subset of alpha = 0.05. In SPSS 19.0 statistical software, SNK in one-way ANOVA was selected to compare the differences of AAB in 10 min, 20 min, 30 min, 40 min, 50 min, and 60 min. The result indicated that there were significant differences (*P* < 0.05) between groups (Table 4), and there was no significant difference between 10 min and 20 min, or among 30 min, 40 min, and 50 min. However, extremely significant differences were observed with the other decoction times (Table 5). This indicated that AAB significantly differs with CR decocting times and could be mainly divided into three periods (time points), namely, the AAB using 10–20 min decoction time was the strongest, then using 30–50 min decoction, and finally 60 min. We concluded that AAB in CR varies with decoction time.

### 3.4. Correlation Analysis of Effective Constituents and AAB In Vitro in CR Using Different Decoction Times

#### 3.4.1. Analysis of Effective Constituents and AAB by SIMCA-PLS

**Figure 6:**
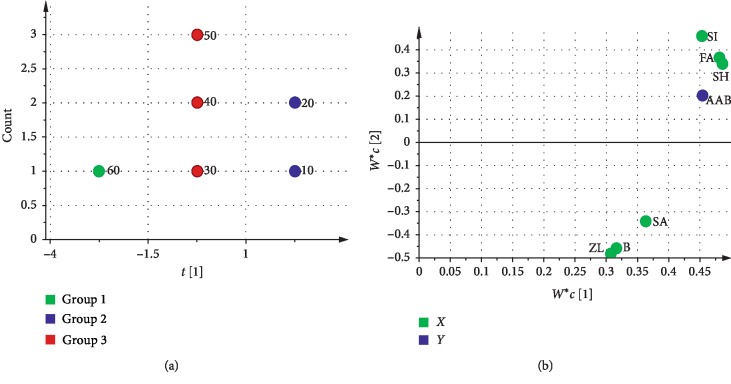
Results of statistical analysis by SIMCA-PLS: (a) dot plot; (b) loading scatter plot.

Based on results of SIMCA-PCA, ferulic acid, senkyunolide I, senkyunolide H, Z-ligustilide, butylphthalide, and senkyunolide A as independent variable (*X*), and AAB as dependent variable (*Y*) were conducted by SIMCA-PLS. The result of SIMCA-PLS indicated that the first two principal components were selected, *R*^2^*X* (cum) was 0.993 and *Q*^2^ (cum) was 0.941. Under the conditions of effective ingredients and AAB, the decoction times were divided into three parts: 10–20 minutes, 30–50 minutes, and 60 minutes (Figure 6(a)). In the loading scatter plot of SIMCA-PLS, six effective components and AAB were divided into two parts: Z-ligustilide, butylphthalide, and senkyunolide A were concentrated, while AAB and senkyunolide I, senkyunolide H, and ferulic acid were concentrated. The result showed that there was a strong correlation among Z-ligustilide, butylphthalide, and senkyunolide A. And there was a strong correlation among senkyunolide I, senkyunolide H, and ferulic acid. Meanwhile, the change rule of AAB was similar to that of senkyunolide I, senkyunolide H, and ferulic acid (Figure 6(b)).

#### 3.4.2. Variables of Pearson Correlation Analysis

The correlations of two variables could be studied by Pearson correlation analysis. In the decoction of CR for 10–60 minutes, vanillin, ferulic acid, senkyunolide I, senkyunolide H, senkyunolide A, butylphthalide, Z-ligustilide, and AAB were selected to conduct Pearson correlation analysis (Table 6). The results indicated that there were significant differences (*P* < 0.05) between effective components, which were ferulic acid and senkyunolide I (correlation was 0.976), ferulic acid and senkyunolide H (correlation was 0.972), senkyunolide I and senkyunolide H (correlation was 0.982), senkyunolide A and butylphthalide (correlation was 0.974), senkyunolide A and Z-ligustilide (correlation was 0.947), butylphthalide and Z-ligustilide (correlation was 0.993), respectively. There were significant differences (*P* < 0.05) between effective components and AAB, which were ferulic acid (correlation was 0.965), senkyunolide I (correlation was 0.973), and senkyunolide H (correlation was 0.999). The results were consistent with SIMCA-PLS.

#### 3.4.3. Multiple Regression Analysis

The relationship may be multivariate linear regression between effective components and AAB; i.e., multicomponent was correlated with AAB at the same time. According to the results of stepwise regression analysis, the *R*^2^ of the model was 1 and the adjusted *R*^2^ was 1, which showed that the model had a good fit (Table 7). In variance analysis, the linear regressions of senkyunolide H and senkyunolide I had statistical significance (*P* < 0.05), and other effective components were of statistical significance (*P* > 0.05) (Table 8). There were correlations between senkyunolide H, senkyunolide I, and AAB. Senkyunolide H (H) was positively correlated with AAB. Senkyunolide I (I) was negatively correlated with AAB, and its expression was AAB = 1.187 *∗* H − 0.199 *∗* I − 0.422 (Tables 9 and 10).

## 4. Conclusion

The contents of effective ingredients are different in CR at different times. With the prolongation of decoction time, effective ingredients possess transformation and degradation, and AAB decreases. The changes of effective ingredients are consistent with AAB in CR decoction: 10 minutes, 20 minutes > 30 minutes, 40 minutes, and 50 minutes > 60 minutes. There are synergistic relationships between effective ingredients; meanwhile, effective ingredients and AAB are correlated. In the analysis of correlation between multicomponent and AAB, senkyunolide I, senkyunolide H, and AAB showed a linear relationship, senkyunolide H was positively correlated, senkyunolide I was negatively correlated, and the other effective ingredients were not correlated with AAB.

## Figures and Tables

**Table 1:** Calibration curves for 9 ingredients.

Compound	Linear	*R* ^2^	Range (*μ*g/ml)	LOD (ng/ml)	LQD (ng/ml)
Vanillin	*Y* = 10387 *X* − 5.4863	0.9997	0.09656–1.4484	4.37	13.52
Ferulic acid	*Y* = 3265.1*X* − 3.468	0.9999	0.06784–1.0176	3.29	9.98
Senkyunolide I	*Y* = 16138*X* − 1.3311	1.0000	0.0904–1.356	4.31	12.90
Senkyunolide H	*Y* = 16330*X* − 1.3378	1.0000	0.0596–0.894	2.10	6.71
Coniferyl ferulate	*Y* = 4648*X* + 3.5724	1.0000	0.1507–2.2608	2.31	6.98
Senkyunolide A	*Y* = 2982*X* + 1.2708	0.9999	0.18224–2.7336	0.41	1.38
Butylphthalide	*Y* = 7455.1*X* + 0.4517	0.9999	0.01119–1.791	1.22	4.01
Z-Ligustilide	*Y* = 3902.7*X* + 4.7909	1.0000	0.2312–3.468	1.47	5.10
Levistilide A	*Y* = 8457*X* − 1.4961	1.0000	0.07944–1.1916	0.75	2.49

**Table 2:** UPLC method validation parameters for the nine components.

Compound	Precision (RSD) (%)	Stability (RSD) (%)	Reproducibility (RSD) (%)
Vanillin	0.32	0.33	0.34
Ferulic acid	0.35	0.35	0.15
Senkyunolide I	0.23	0.39	0.18
Senkyunolide H	0.34	0.34	0.34
Coniferyl ferulate	0.20	0.20	0.09
Senkyunolide A	0.13	0.10	0.09
Butylphthalide	0.15	0.16	0.16
Z-Ligustilide	0.22	0.25	0.12
Levistilide A	0.26	0.19	0.24

**Table 3:** Contents of nine constituents at different decoction times (mg/g, *n* = 3).

*t* (min)	Vanillin (%)	Ferulic acid (%)	Senkyunolide I (%)	Senkyunolide H (%)	Coniferyl ferulate (%)	Senkyunolide A (%)	Butylphthalide (%)	Z-Ligustilide (%)	Levistilide A (%)
0	0.14 ± 0.69	1.34 ± 0.79	1.54 ± 0.42	0.28 ± 0.44	5.94 ± 1.32	31.47 ± 1.38	0.47 ± 1.36	14.47 ± 1.21	0.02 ± 1.27
10	0.13 ± 1.22	4.19 ± 1.35	1.73 ± 0.81	0.37 ± 0.38	0.00	13.61 ± 1.21	0.16 ± 1.38	1.03 ± 0.98	0.00
20	0.12 ± 1.31	4.25 ± 0.77	1.74 ± 0.38	0.37 ± 0.29	0.00	10.64 ± 0.98	0.12 ± 1.69	0.44 ± 1.35	0.00
30	0.12 ± 1.65	3.97 ± 0.28	1.69 ± 1.12	0.35 ± 0.87	0.00	6.00 ± 0.77	0.08 ± 1.47	0.00	0.00
40	0.12 ± 1.69	3.96 ± 0.54	1.70 ± 1.21	0.34 ± 0.78	0.00	5.95 ± 1.26	0.08 ± 2.19	0.00	0.00
50	0.12 ± 0.89	3.84 ± 0.39	1.71 ± 1.33	0.34 ± 0.98	0.00	6.00 ± 1.39	0.08 ± 1.32	0.00	0.00
60	0.12 ± 1.27	3.12 ± 0.41	1.40 ± 1.29	0.28 ± 1.22	0.00	5.01 ± 1.27	0.08 ± 1.18	0.00	0.00

**Table 4:** ANOVA.

Source	Sum of squares	df	Mean square	*F*	Sig.
Group	0.108	5	0.022	41.711	0.000
Total	0.114	17			

**Table 5:** Student–Newman–Keuls^a^.

Group	*N*	Subset for alpha = 0.05		
		1	2	3
60 min	3	0.248		
50 min	3		0.389	
40 min	3		0.392	
30 min	3		0.424	
20 min	3			0.476
10 min	3			0.479
Sig.		1.000	0.194	0.873

**Table 6:** Correlation coefficients of Pearson correlation analysis.

Variables	Vanillin	Ferulic acid	Senkyunolide I	Senkyunolide H	Senkyunolide A	Butylphthalide	Z-Ligustilide	AAB
Vanillin	—	−0.540	−0.395	−0.466	0.669	0.795	0.859	−0.482
Ferulic acid	—	—	0.976^*∗∗*^	0.972^*∗∗*^	−0.385	−0.548	−0.564	0.965^*∗∗*^
Senkyunolide I	—	—	—	0.982^*∗∗*^	−0.364	−0.503	−0.499	0.973^*∗∗*^
Senkyunolide H	—	—	—	—	−0.319	−0.483	−0.495	0.999^*∗∗*^
Senkyunolide A	—	—	—	—	—	0.974^*∗∗*^	0.947^*∗*^	−0.313
Butylphthalide	—	—	—	—	—	—	0.993^*∗∗*^	−0.481
Z-Ligustilide	—	—	—	—	—	—	—	−0.497
AAB	—	—	—	—	—	—	—	—

^*∗*^ *P* < 0.05; ^*∗∗*^*P* < 0.01.

**Table 7:** Model summary.

Model	*R*	*R* ^2^	Adjusted *R*^2^	Std. error of the estimate
1	1.000^a^	1	1	0.0010433

^a^Predictors: (constant), senkyunolide H, and senkyunolide I.

**Table 8:** ANOVA.

Model		Sum of squares	Df	Mean square	*F*	Sig.
1	Regression	0.036	2	0.018	16575.747	0.000^a^
	Residual	0	3	0		
	Total	0.036	5			

^a^Predictors: (constant), senkyunolide H, and senkyunolide I.

**Table 9:** Coefficients^a^.

Variables	Unstandardized coefficients	Standardized coefficients		*t*	Sig.
	*B*	Std. error	Beta		
(Constant)	−0.422	0.007		−63.728	0
Senkyunolide H	3.044	0.045	1.187	67.199	0
Senkyunolide I	−0.13	0.012	−0.199	−11.244	0.002

^a^Dependent variable: AAB.

**Table 10:** Excluded variables^a^.

Model	Beta	*t*	Sig.	Partial correlation	Collinearity statistics tolerance
Vanillin	0.006^a^	0.799	0.508	0.492	0.622
Ferulic acid	0.011^a^	0.183	0.872	0.128	0.012
Senkyunolide A	−0.001^a^	−0.091	0.936	−0.064	0.195
Butylphthalide	0.001^a^	0.1	0.929	0.071	0.264
Z-Ligustilide	0.003^a^	0.229	0.84	0.16	0.302

^a^Predictors: (constant), senkyunolide H, and senkyunolide I.

## Data Availability

The data used to support the findings of this study are available from the corresponding author upon request.

## References

[1] Chinese Pharmacopoeia Commission (2015). *Chinese Pharmacopoeia*.

[2] Naito T., Kubota K., Shimoda Y. (1995). Effects of constituents in a Chinese crude drug, Ligusticum chuanxiong Rhizoma on vasocontraction and blood viscosity. *Natural Medicines*.

[3] Chen C., Chen J., Wu H. (2011). Identification of key constituents in volatile oil of Ligusticum chuanxiong based on data mining approaches. *Pharmaceutical Biology*.

[4] Zhao X., Ma T., Zhang C. (2015). Simultaneous determination of senkyunolide I and senkyunolide H in rat plasma by LC-MS: application to a comparative pharmacokinetic study in normal and migrainous rats after oral administration of Chuanxiong Rhizoma extract. *Biomedical Chromatography*.

[5] Li S. L., Chan S. S., Lin G. (2003). Simultaneous analysis of seventeen chemical ingredients of Ligusticum Chuanxiong by on-line high performance liquid chromatograph-diode array detector-mass spectrometry. *Planta Medica*.

[6] Yan R., Li S. L., Chuang H. S. (2003). Simultaneous quantification of 12 bioactive constituents of *Ligusticum Chuanxiong* Hort. By high-performance liquid chromatography. *Journal of Pharmaceutical and Biomedical Analysis*.

[7] Kuang X., Yao Y., Du J. R., Liu Y. X., Wang C. Y., Qian Z. M. (2006). Neuroprotective role of Z-ligustilide against forebrain ischemic injury in ICR mice. *Brain Research*.

[8] Peng H.-Y., Du J.-R., Zhang G.-Y. (2007). Neuroprotective effect of Z-ligustilide against permanent focal ischemic damage in rats. *Biological and Pharmaceutical Bulletin*.

[9] Cao Y.-X., Zhang W., He J.-Y., He L.-C., Xu C.-B. (2006). Ligustilide induces vasodilatation via inhibiting voltage dependent calcium channel and receptor-mediated Ca2+ influx and release. *Vascular Pharmacology*.

[10] Chan S. S.-K., Cheng T.-Y., Lin G. (2007). Relaxation effects of ligustilide and senkyunolide A, two main constituents of Ligusticum chuanxiong, in rat isolated aorta. *Journal of Ethnopharmacology*.

[11] Qi H., Siu S. O., Chen Y. (2010). Senkyunolides reduce hydrogen peroxide-induced oxidative damage in human liver HepG2 cells via induction of heme oxygenase-1. *Chemico-Biological Interactions*.

[12] Zhu M., Tang Y., Duan J.-a. (2010). Roles of paeoniflorin and senkyunolide I in SiWu decoction on antiplatelet and anticoagulation activities. *Journal of Separation Science*.

[13] Yang L.-C., Li J., Xu S.-F. (2015). L-3-n-butylphthalide promotes neurogenesis and neuroplasticity in cerebral ischemic rats. *CNS Neuroscience and Therapeutics*.

[14] Li Q., Cheng Y., Bi M. (2015). Effects of N-butylphthalide on the activation of Keap1/Nrf-2 signal pathway in rats after carbon monoxide poisoning. *Environmental Toxicology and Pharmacology*.

[15] Jia J., Wei C., Liang J. (2015). The effects of DL-3-n-butylphthalide in patients with vascular cognitive impairment without dementia caused by subcortical ischemic small vessel disease: a multicenter, randomized, double-blind, placebo-controlled trial. *Alzheimers and Dementia*.

[16] Hsieh M.-T., Tsai F.-H., Lin Y.-C., Wang W.-H., Wu C.-R. (2002). Effects of ferulic acid on the impairment of inhibitory avoidance performance in rats. *Planta Medica*.

[17] Naito T., Iketani Y., Kubota K. (1995). Vasodilators containing coniferyl ferulate and phthalide dimers of Cnidium officinale.

[18] Puzio K., Delépée R., Vidal R., Agrofoglio L. A. (2013). Combination of computational methods, adsorption isotherms and selectivity tests for the conception of a mixed non-covalent-semi-covalent molecularly imprinted polymer of vanillin. *Analytica Chimica Acta*.

[19] Li S.-L., Yan R., Tam Y.-K., Lin G. (2007). Post-harvest alteration of the main chemical ingredients in *Ligusticum chuanxiong* Hort. (Rhizoma chuanxiong). *Chemical and Pharmaceutical Bulletin*.

[20] Lu G.-H., Chan K., Leung K., Chan C.-L., Zhao Z.-Z., Jiang Z.-H. (2005). Assay of free ferulic acid and total ferulic acid for quality assessment of Angelica sinensis. *Journal of Chromatography A*.

[21] Tiemann F., Haarmann W. (2010). Ueber das coniferin und seine umwandlung in das aromatische princip der vanille. *European Journal of Inorganic Chemistry*.

[22] Zuo A. H., Wang L., Xiao H. B. (2012). Study on degradation products of senkyunolide A and senkyunolide I. *Chinese Traditional and Herbal Drugs*.

[23] Yao Y. X., Hua F., Hong Y. L. (2017). Bioassay of antiplatelet aggregation bioactivity in Chuanxiong Rhizoma with related Chinese patent drugs. *Chinese Traditional and Herbal Drugs*.

[24] He C.-Y., Wang S., Feng Y. (2012). Pharmacokinetics, tissue distribution and metabolism of senkyunolide I, a major bioactive component in Ligusticum chuanxiong Hort. (Umbelliferae). *Journal of Ethnopharmacology*.

[25] Yan R., Ko N. L., Li S.-L., Tam Y. K., Lin G. (2008). Pharmacokinetics and metabolism of ligustilide, a major bioactive component in rhizoma chuanxiong, in the rat. *Drug Metabolism and Disposition*.

[26] Tang J., Chen C., Yu Y. (2009). Pharmacological activities of Z-ligustilide and metabolites in rats. *Journal of Sichuan University*.

